# Evaluation of Recombinase Polymerase Amplification assay for monitoring parasite load in patients with kala-azar and post kala-azar dermal leishmaniasis

**DOI:** 10.1371/journal.pntd.0011231

**Published:** 2023-04-19

**Authors:** Madhurima Roy, Arianna Ceruti, Rea Maja Kobialka, Sutopa Roy, Deblina Sarkar, Ahmed Abd El Wahed, Mitali Chatterjee

**Affiliations:** 1 Dept. of Pharmacology, Institute of Post Graduate Medical Education and Research (IPGME&R), Kolkata, India; 2 Institute of Animal Hygiene and Veterinary Public Health, Leipzig University, Leipzig, Germany; London School of Hygiene and Tropical Medicine, UNITED KINGDOM

## Abstract

**Background:**

The potential reservoirs of visceral leishmaniasis (VL) in South Asia include asymptomatic and relapsed cases of VL, along with patients with post kala-azar dermal leishmaniasis (PKDL). Accordingly, accurate estimation of their parasite load is pivotal for ensuring disease elimination, presently targeted for 2023. Serological tests cannot accurately detect relapses and/or monitor treatment effectiveness, and therefore, parasite antigen/nucleic acid based detection assays remain the only viable option. An excellent option is the quantitative polymerase chain reaction (qPCR) but the high cost, technical expertise and time involved precludes its wider acceptability. Accordingly, the recombinase polymerase amplification (RPA) assay operated in a mobile suitcase laboratory has emerged not simply as a diagnostic tool for leishmaniasis but also to monitor the disease burden.

**Methodology/Principal findings:**

Using total genomic DNA isolated from peripheral blood of confirmed VL cases (n = 40) and lesional biopsies of PKDL cases (n = 64), the kinetoplast-DNA based qPCR and RPA assay was performed and parasite load expressed as Cycle threshold (Ct) and Time threshold (Tt) respectively. Using qPCR as the gold standard, the diagnostic specificity and sensitivity of RPA in naïve cases of VL and PKDL was reiterated. To assess the prognostic potential of the RPA, samples were analyzed immediately at the end of treatment or ≥6 months following completion of treatment. In cases of VL, the RPA assay in terms of cure and detection of a relapse case showed 100% concordance with qPCR. In PKDL following completion of treatment, the overall detection concordance between RPA and qPCR was 92.7% (38/41). At the end of treatment for PKDL, 7 cases remained qPCR positive, whereas RPA was positive in only 4/7 cases, perhaps attributable to their low parasite load.

**Conclusions/Significance:**

This study endorsed the potential of RPA to evolve as a field applicable, molecular tool for monitoring parasite load, possibly at a point of care level and is worthy of consideration in resource limited settings.

## Author summary

Visceral leishmaniasis (VL) or kala-azar caused by the digenetic parasite *Leishmania donovani* is a fatal form of leishmaniasis, unless treated. In South Asia, approximately 10–20% of apparently cured cases of VL develop a dermal sequela termed post kala-azar dermal leishmaniasis (PKDL). It presents with macular or papulonodular lesions that harbour *Leishmania* parasites. Importantly, these cases of PKDL in addition to asymptomatic and relapsed VL cases serve as mobile disease reservoirs and sustain disease transmission. In macular PKDL, that presently comprise over 50% of cases, the diagnosis is particularly challenging as parasites are often not detectable by microscopy. Accordingly, development of strategies for molecular diagnosis is one of the major foci of the ongoing leishmaniasis elimination programme targeted for 2023, that aims to decrease the burden to <1/10,000 cases of VL at the block/zilla level. Serological tests have limited applicability when it is necessary to distinguish present from past infection of VL, and in cases of PKDL when the positivity maybe due to a past history of VL. An excellent option is the quantitative polymerase chain reaction (qPCR) but limitations for its wider field applicability include the high cost, technical expertise and time factor. Accordingly, an urgent need exists for availability of field applicable molecular tests. The RPA tested in this study could well be the desired game-changer as it provided accurate diagnosis of VL and PKDL, can be performed in a primary health centre, and demonstrated the potential to be a monitoring tool for parasite load after treatment, that eventually can accelerate achieving the end-goal of elimination of leishmaniasis.

## Introduction

Leishmaniasis, a neglected tropical disease of poverty is caused by the parasitic protozoan of the genus *Leishmania*. The disease spectrum ranges from a self-healing cutaneous lesion (cutaneous leishmaniasis, CL) to the fatal visceral form (visceral leishmaniasis, VL or kala-azar). Additionally, in South Asia (mainly India, Bangladesh and Nepal), a small proportion (approximately 10–20%) of apparently cured VL cases develop a dermal sequela called post kala-azar dermal leishmaniasis, PKDL [1,2] that present with a combination of macules, papules and nodules (polymorphic) or hypomelanotic lesions (macular). In view of PKDL cases harbouring parasites in their skin lesions, which are accessible to the sand fly for a blood meal, they are considered as disease transmitters and therefore hold immense epidemiological significance [3]. With active surveillance initiated from 2015 onwards as a component of the ongoing kala-azar elimination programme (KAEP), a substantial proportion of new PKDL cases have been identified and importantly, a large proportion of macular cases were unearthed [4–6].

The current target to eliminate kala-azar from South Asia has been set at 2023 and sustainability will be monitored till 2030 with a target of <1 case within 10,000 population at district level in Nepal and sub-district level in India and Bangladesh [7]. This highlights the urgent need for availability of techniques for early detection of the parasite load and monitoring therapeutic effectiveness. Presently, emphasis of the KAEP is to address the last mile challenges to accelerate VL elimination. Accordingly, areas of focus include early case detection, vector control, and identification of new foci as also introduction of molecular approaches for monitoring chemotherapeutic responses [6].

For effective case management, tests should not only accurately detect cases of VL and PKDL but also be able to assess the impact of treatment i.e., be a test-of-cure [8]. The gold standard for diagnosis of visceral leishmaniasis is the microscopic demonstration of amastigotes (Leishman Donovan, LD bodies) in a spleen or bone marrow aspirate. For PKDL, gold standard diagnosis is observation of the amastigotes in slit skin smears. However, being an invasive procedure, the diagnosis of VL has since been replaced by serological tests (World Health Organisation, WHO guidelines [https://www.who.int/news-room/fact-sheets/detail/leishmaniasis, accessed on 29^th^ July, 2022]). The confirmatory diagnosis of PKDL remains challenging, especially if there is no defined history of VL, and if the lesions are macular as LD bodies are practically impossible to find, as also in cases where the lesions are not distinguishable from leprosy [9]. Furthermore, the rK39 (recombinant K39 antigen) strip test can falter, as positivity could be due to a past history of VL. Accordingly, in the absence of a defined test, an algorithm for diagnosis of PKDL has been proposed [https://www.who.int/publications/i/item/9789241505215, accessed on 6^th^ August, 2022]. Taken together, the current challenges for management of VL and PKDL include lack of availability of objective tools for (a) assessment of cure and diagnosis of relapsed VL cases, along with (b) confirmatory diagnosis and monitoring treatment effectiveness in PKDL [10]. The macular form of PKDL was earlier considered as a minor component of the disease. However, with active surveillance, the proportion of macular PKDL has increased to nearly 50% [5]. Importantly, as macular PKDL harbour a limited number of parasites that cannot be detected by microscopic examination, there is a need for molecular tools to be established and introduced for better diagnosis and monitoring of PKDL. To address these limitations, molecular tools like the quantitative real-time polymerase chain reaction (qPCR) have been efficiently employed using specific targets for amplification i.e. kinetoplast DNA (kDNA) for diagnostics and monitoring patients with VL and PKDL [10 and references therein]. Moreover, quantification of parasite load by qPCR targeting the kDNA has proved to be an effective approach for monitoring and assessing cure in patients with PKDL [4]. However, as these tests require a well-equipped laboratory, highly-trained personnel, maintenance of cold chain for reagents, their applicability in resource limited settings (e.g., primary health-care facilities) becomes limited. An emerging alternative option translatable to a field scenario is the use of loop mediated isothermal amplification (LAMP) assay for VL and PKDL [10 and references therein]. Therefore, if elimination of kala-azar from South Asia is to become a reality, the availability of field applicable, low cost molecular detection tools is top priority as it will be able to objectively quantify the parasite load [6–8].

The recombinase polymerase amplification (RPA) assay is an isothermal amplification system wherein the reagents are cold chain independent and can be kept at 38–40°C for up to three months without the assay performance being impacted [11]. The RPA assay has been applied for detection of respiratory syndrome coronavirus, avian influenza A (H7N9) virus, dengue, ebola virus, monkey-pox virus, feline coronavirus and rabies virus [12–18]. In South Asia, the diagnostic performance of the RPA involving clinical samples from VL/PKDL/CL cases has been validated in India, Bangladesh, Nepal and Sri Lanka [19–22], but its applicability as a prognostic molecular tool has not been addressed. The field deployable RPA assay has also been validated according to standards for reporting of diagnostic accuracy studies (STAARD) guidelines, endorsing its applicability over traditional laboratory based molecular approaches [21]. Accordingly, this study aimed to evaluate the RPA as a tool for monitoring the parasite load with a view to translate its application into a field setting.

## Materials and methods

### Ethics statement

The study was approved by the Institutional Ethics committee of Institute of Post Graduate Medical Education & Research (IPGME&R/IEC/2021/273) for use of freshly collected and repurposed samples stored in the repository of IPGME&R, Kolkata, India. Written informed consent was obtained from all patients or their legally accepted representative.

### Study population

**Fig 1:**
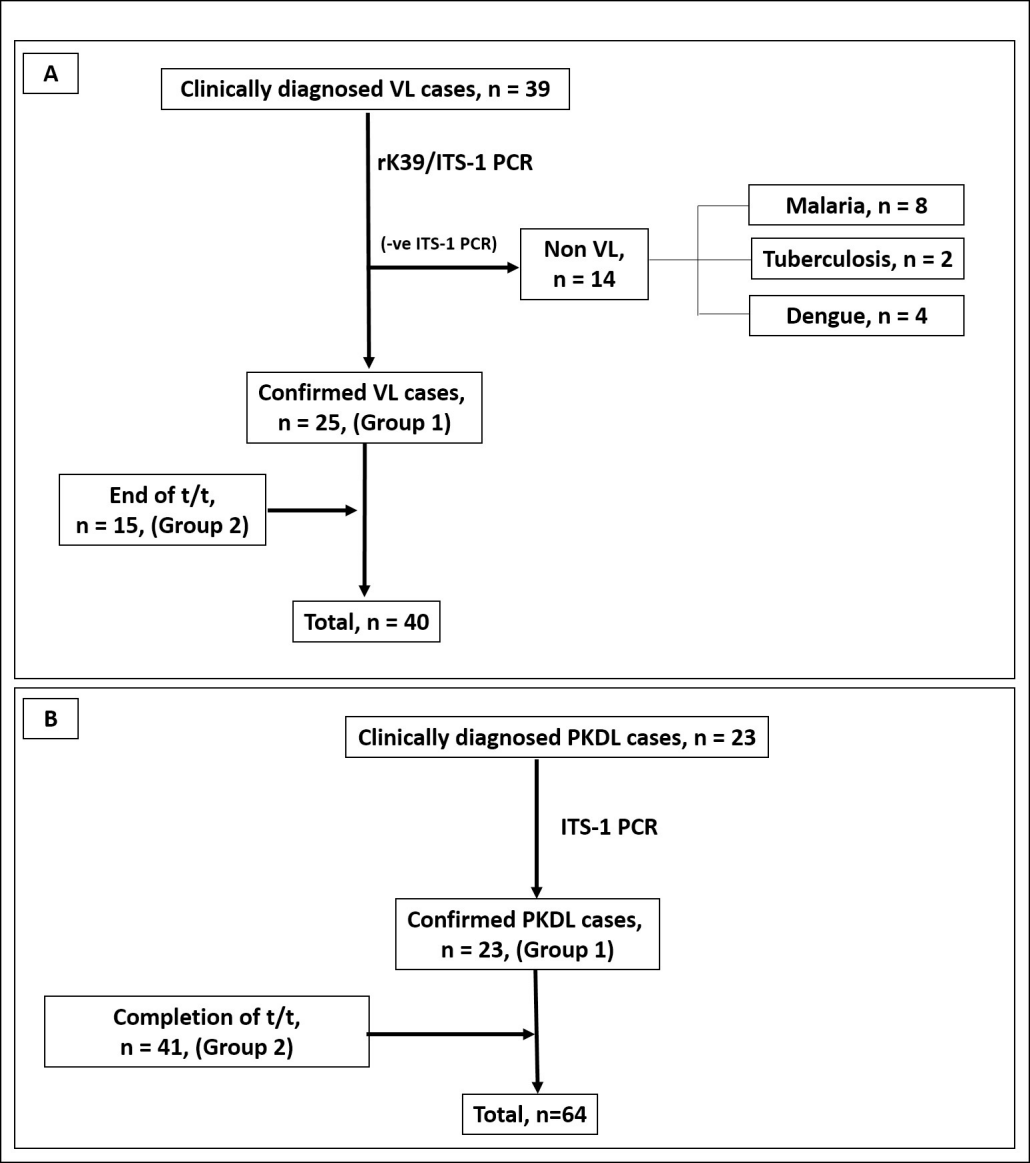
Schematic diagram indicating enrolment of patients with visceral leishmaniasis (VL,**A**) and post kala-azar dermal leishmaniasis (PKDL, **B**) that were tested by qPCR and RPA as described in materials and methods. Patients with VL/PKDL were recruited at disease presentation (Group 1) or at completion of treatment (Group 2). The diagnostic potential of the RPA assay was reiterated with Group 1, while the prognostic potential was evaluated with Group 2. t/t: treatment.

The study initially included suspected patients with VL (n = 39), who were admitted to the Department of Tropical Medicine, School of Tropical Medicine (STM), Kolkata (**Table 1**). Their initial diagnosis was empirically based on clinical features such as fever for more than two weeks, hepatosplenomegaly and residing/visiting an area endemic for VL. Alongside, serological diagnosis involved the rK39 (InBios International, Inc. WA, USA) strip test that detected the highly conserved K39 epitope in visceralizing species of *Leishmania spp* [23], enabling detection of visceral leishmaniasis (kala-azar) antibodies in human serum. Accordingly, the diagnosis of VL was confirmed in 25/39 cases in peripheral blood by rK39 strip test and Internal Transcribed Spacer 1-PCR (ITS1-PCR) and were considered as ‘naïve’ cases, Group 1 (**Fig 1A**). These tests (rK39 and ITS1-PCR) were performed as patient associated information in a hospital setting, these results have not been incorporated into this study. Diagnosis by ITS1-PCR involved amplification using *Leishmania*-specific primers LITSR (5′-CTGGATCATTTTCCGATG-3′) and L5.8S (5′-TGATACCACTTATCGCACTT-3′) using Red Taq polymerase in a Master cycler (Eppendorf, Hamburg, Germany) [4]. The positive control was DNA sourced from a *Leishmania donovani* strain MHOM/IN/1983/AG83. The remaining 14/39 ITS1-PCR negative cases, had clinical symptoms suggestive of VL, i.e., fever and hepatosplenomegaly, but negative for serological tests (rK39) and ITS1-PCR and were categorised as ‘non VL’ (**Fig 1A)**. Non VL cases were included in the study population to assess any cross reactivity by RPA. Additionally, 15 cases of VL who had completed treatment (> 6months, Group 2) were included; accordingly, a total of 40 cases of VL (Group 1, n = 25 and Group 2, n = 15) were analysed (**Fig 1A)**. Treatment received by patients in Group 2 was either a single dose of Liposomal Amphotericin B, (LAmB, 10 mg/kg b.w., n = 13) or Miltefosine (28 days, if < 25 kg b.w., 50 mg p.o./daily and if >25 kg b.w., 100 mg p.o./daily, n = 2). Heparinised blood was collected from all VL cases.

**Table 1:** Study population to assess the performance of qPCR vs. RPA in cases of VL.

Clinical features	Patients with VL (n = 40)		non VL (n = 14)
	Group 1 Presentation (n = 25)	Group 2 Post t/t (n = 15)	• malaria (n = 8) • hepatic military tuberculosis (n = 2) • dengue (n = 4)
*Age, years	35.00 (18.50–49.00)	40.00 (24.00–47.00)	23.00 (17.00–38.00)
Sex (Male: Female)	16:9	7:8	6:8
*Liver Size (cm)	4.50 (2.47–6.00)	#4.00 (n = 1)	6.0 (3.0–9.0)
*Spleen Size (cm)	13.50 (9.00–17.45)	#7.00 (n = 1)	17.85 (6.12–20.25)
*Hb count (g/dL)	7.75 (7.43–9.35)	#10.60 (n = 1)	6.0 (5.0–10.0)
*Duration of fever (months)	5.00 (2.50–6.00)	#0.3 (n = 1)	1.5 (0.65–15.0)
Past history of VL	No	Yes	No

Peripheral blood was collected from suspected cases of VL as described in materials and methods. The sizes of liver and spleen were based on manual palpation by the attending physician

*Values are stated as median (IQR), VL: visceral leishmaniasis, non VL: patients suspected with VL but tested negative for rK39/ITS1-PCR, post t/t: upon completion of treatment with Liposomal Amphotericin B or Miltefosine as described in materials and methods. ^#^features of relapse (hepatosplenomegaly and fever for more than 2 weeks) was present in one post treatment case.

Patients clinically diagnosed with PKDL (n = 23, **S1 Fig**) were recruited from different sites, namely Dermatology outpatient departments of School of Tropical Medicine/ Calcutta Medical College/ Institute of Postgraduate Medical Education and Research, Kolkata, West Bengal or during active field surveys conducted in endemic districts of West Bengal (Malda, Dakshin Dinajpur, Murshidabad, and Birbhum) and Jharkhand (Dumka, Pakur, Sahibganj, Godda, **Table 2**). In these field studies, a camp approach was used, wherein initially a house-to-house survey was conducted by first-line health care workers i.e. kala-azar technical supervisors. An empirical diagnosis of PKDL was made based on the WHO algorithm, i.e. clinical features strongly suggestive of PKDL, a prior history of VL, rK39 positivity and/or if they resided in an area endemic for VL, and were then examined by medical personnel at medical camps [5]. Following infiltration of lignocaine hydrochloride (2%), a 4-mm skin biopsy was collected from the suspected cases, their diagnosis was confirmed by ITS1-PCR and were considered as naïve (n = 23, Group 1, **Fig 1B**). These cases included patients with hypopigmented lesions and were termed macular PKDL, and patients with a miscellany of papules, nodules, macules, and/or plaques were considered as polymorphic PKDL [24]. In addition, PKDL cases were enrolled that had received Miltefosine (Group 2, n = 41, **Fig 1B and Table 2**), for 12 weeks, daily, 50 mg p.o., if <25 kg b.w., and if >25 kg b.w., 100 mg p.o. as per national guidelines [National Vector Borne Disease Control Programme (Guidelines for treatment of post-kala-azar dermal leishmaniasis. Available at: https://nvbdcp.gov.in/Doc/PKDL-Guidelines-220512.pdf) accessed on 10^th^ May, 2022]. Additionally, to confirm absence of cross-reactivity, confirmed cases of leprosy (n = 10) were included (**Table 2**). None of the patients included in the study for VL and PKDL suffered from any co-infection(s), as confirmed by the treating physician, and pregnant women were excluded. Accordingly, patients of VL or PKDL in Group 1 were tested for confirming the diagnostic potential of the RPA assay, while patients of VL/PKDL in Group 2 who had completed treatment were tested for prognostic potential of the RPA assay.

**Table 2:** Study population to assess performance of qPCR vs. RPA in PKDL.

Clinical features	Patients with PKDL (n = 64)			leprosy (n = 10)
	Group 1 Presentation (n = 23)	Group 2 Post treatment (n = 41)		
		End of 12 weeks treatment (n = 8)	Follow up, >6months (n = 33)	
*Age, years	17.00 (9.87–29.25)	15.50 (13.25–24.25)	23.00 (16.00–35.00)	41.00 (30.00–48.00)
Sex (Male: Female)	11:12 (1.00:1.10)	4:4 (1.00:1.00)	21:12 (1.75:1.00)	8:2 (4.00:1.00)
History of VL	23/23 = 100%	8/8 = 100%	33/33 = 100%	NA
Lesion type (Macular: Polymorphic)	14:9 (1.6:1.0)	2:6 (1.0:3.0)	22:11 (2.0:1.0)	Hypopigmented/ Hypoesthetic patches all over body
*Lag period, years	5.00 (3.00–10.00)	3.00 (2.25–9.00)	8.00 (4.00–11.50)	NA
*Disease duration, years	3.00 (2.00–7.50)	1.00 (1.00–2.50)	3.50 (1.75–7.25)	1.20 (1.00–2.00)

Skin biopsies were collected from suspected cases of PKDL as described in materials and methods.

*Values are stated as median (IQR), Lag period was the interval between cure from VL and onset of features of PKDL. Disease duration is the time between the onset of PKDL and inclusion in this study. PKDL post t/t: following completion of treatment with Miltefosine, as described in materials and methods, NA: not applicable.

### Molecular standards

DNA was used from two reference strains of *L*. *donovani* (MHOM/IN/80/DD8, WHO collaborating centre for leishmaniasis, Laboratorio de Referencia e Investigación en Parasitología Centro Nacional de Microbiología, Instituto De Salud Carlos III, Spain and MHOM/IN/83/AG83). A standard curve was generated with serially diluted parasite DNA for amplification of target kDNA by qPCR and RPA. To establish the limit of detection of the assays, a defined number of *Leishmania* parasites ranging from 10^4^ to 10^0^ genomes equivalent copies of parasite DNA/reaction were used.

### Detection of parasite load by Real-time quantitative Polymerase Chain Reaction (qPCR)

DNA was extracted according to the manufacturer’s instructions (QIAmp DNA mini kit, Qiagen, Hilden, Germany) from (a) peripheral blood of patients suspected/diagnosed with VL and (b) skin biopsies in phosphate-buffered saline (20 mM, pH 7.4, PBS) from patients suspected/diagnosed with PKDL. The skin biopsies were then excised into small pieces, and DNA eluted in 50 μL of DNA elution buffer. The concentration of double stranded DNA was measured in a Nanodrop One Microvolume UV-Vis Spectrophotometer (Thermo Fischer Scientific, MA, USA); the yield ranged from 90–150 ng/μL and ratio of absorbances A260/280 was 1.7–1.8. Additionally, a human specific *18s* semi-quantitative PCR was performed on randomly selected DNA samples from peripheral blood of VL patients (n = 5) and skin biopsies of patients with PKDL (n = 5) to confirm the quality of the extracted DNA, especially for parasite negative samples (**S2 Fig**).

Real-time PCR was performed using TaqMan based detection employing LightCycler Multiplex DNA Master (Roche, Basel, Switzerland). The primer sequences were (kDNA forward primer: 5´-CTTTTCTGGTCCTCCGGGTAGG, reverse primer: 5´-CCACCCGGCCCTATTTTACACCAA-3´) with probe (5´-6FAM-TTTTCGCAGAACGCCCCTACCCGC-BBQ-3´). Briefly, DNA (5 μL) was added to a 15 μL reaction mixture containing TaqMan Master Mix (100 nM of each primer and 50 nM of probe). The thermal cycling conditions comprised an initial heating step of 95°C for 1 min, 45 cycles of a denaturation step at 95°C for 10 secs, a combined annealing and extension step of 60°C for 45 secs and finally a single cooling step of 40°C for 30 secs. *Leishmania donovani* molecular standard (MHOM/IN/80/DD8) were included in each run as a positive control while the negative control was nuclease free water (non-template control, NTC). The cut-off was set at a Cycle threshold (Ct) value >30, as it corresponded to a parasite number ≤10 (ABI StepOnePlus Real-Time PCR System Thermal Cycler).

### Detection of parasite load by Recombinase Polymerase Amplification (RPA) assay

The RPA was performed with a total reaction volume of 50 μL using TwistAmp exo kits according to the manufacturer’s instructions (Twist Dx, Cambridge, UK). The RPA primers and exo probe combination were employed based on a previous design [25]. All oligonucleotides were produced by TibMolBiol (Berlin, Germany). Briefly, the reaction was performed with RPA exo-probe oligo mix (13 μL, 21 pMol for each primer and 6 pMol for the probe), magnesium acetate (2.50 μL, 280 mM) and rehydration buffer (29.5 μL). All components were added on the lid of each tube of the eight-tube RPA strips leaving the pellet containing dried enzyme undissolved. DNA template (5 μL) was added into each lid to make the reaction volume 50 μL. The reaction tubes were closed, mixed thoroughly and then placed in the ESEQuant Tube Scanner (Qiagen, Lake Constance, Germany) where heating at a constant temperature of 40°C and real-time monitoring of fluorescence was conducted for 15 minutes. After 230 secs., a brief mixing and a pulse spin was done, thereafter the tubes were returned into the device. The emitted fluorescence signals were measured at 20 secs. scan intervals. Fluorescence was detected in the FAM channel (470 nm excitation and 520 nm emission). The threshold time (Tt) was determined by combining threshold and signal loop analysis (1st derivative analysis) in terms of mV/min using the ESEQuant Tube Scanner software version 1.0 [25]. The total time for the reaction was 15 min. Each run included one *Leishmania donovani* molecular standard as positive control and one reaction mix with molecular grade water as NTC. In future, a housekeeping 18*s* internal control may be considered for RPA assay along with positive and negative controls for each run.

### Evaluation of cure positive predictive (PPV%) and cure negative predictive values (NPV%)

To determine the proportion of true negative and true positive predictions in RPA, with qPCR as the gold standard, the cure positive predictive value (PPV%) and negative predictive value (NPV%) was measured. The ‘true negative’ or ‘true positive’ were cases that were both qPCR and RPA negative or positive respectively. The ‘false negative’ cases were those that were qPCR positive but RPA negative, while the ‘false positive’ cases were qPCR negative, but RPA positive. Accordingly, the cure PPV(%) and NPV(%) were calculated as [true negative/(true negative + false negative)]x100% and [true positive/(true positive + false positive)]x100% respectively.

### Statistical analysis

Data were expressed as median (interquartile range [IQR]) or mean ± SD (standard deviation) with CV% (coefficient of variation). Normal distribution of the data sets was analysed by Shapiro-Wilk test. Correlation was calculated using both Pearson and Spearman rank correlations, using GraphPad Prism software version 8.4.2 (GraphPad Software, La Jolla, California, USA); p<0.05 was considered statistically significant.

## Results

### Study population

The study included 40 cases of VL, of which 25 were confirmed by ITS-1 PCR (Group 1) and an additional 15 were included whose samples were available only on completion of treatment (Group 2, **Fig 1A**). At disease presentation, fever was consistently present along with hepatosplenomegaly (**Table 1**) and was comparable with the non VL group (n = 14), that included cases which were finally accorded a diagnosis of malaria (n = 8), hepatic military tuberculosis (n = 2) or dengue (n = 4). Ideally, the prognostic potential of an assay should be performed by longitudinal monitoring. In this study, many cases were collected during the COVID-19 pandemic and it posed logistical limitations in obtaining paired samples i.e. at presentation and upon completion of 12 months treatment.

In case of PKDL, irrespective of whether they were recruited at disease presentation (Group 1, **Fig 1B and Table 2**) or at the end of treatment (Group 2, **Fig 1B and Table 2**), they were age and gender matched. The proportion of macular and polymorphic cases of PKDL was also comparable. All patients gave a past history of VL, demonstrated a considerably long lag period i.e. gap between manifestations of PKDL following their completion of treatment for VL, and the disease duration of PKDL in Group 1 was comparable with the follow up cases (**Table 2**).

In the PKDL post treatment group (Group 2), there were 8/41 cases whose biopsies were collected at the end of 12 weeks treatment, and majority had polymorphic (6/8, 75%) lesions. Amongst these 6 polymorphic cases, lesions persisted in 4/6 cases (66.6%). The remaining 2/8 cases were macular and both continued to demonstrate lesions. Amongst the 33/41 cases that reported at least 6 months after completion of treatment, they included 11/33 (33.3%) with polymorphic lesions; amongst them, 10/11 (90.9%) continued to show lesions, despite presenting ≥6 months post-treatment. The remaining 22/33 (66.6%) were macular cases and lesions persisted in 7/22 cases (31.8%). This evaluation was performed independently by a dermatologist based on images provided.

### Determination of cut-off threshold for cycle number and time in min. with *L*. *donovani* standards

**Fig 2:**
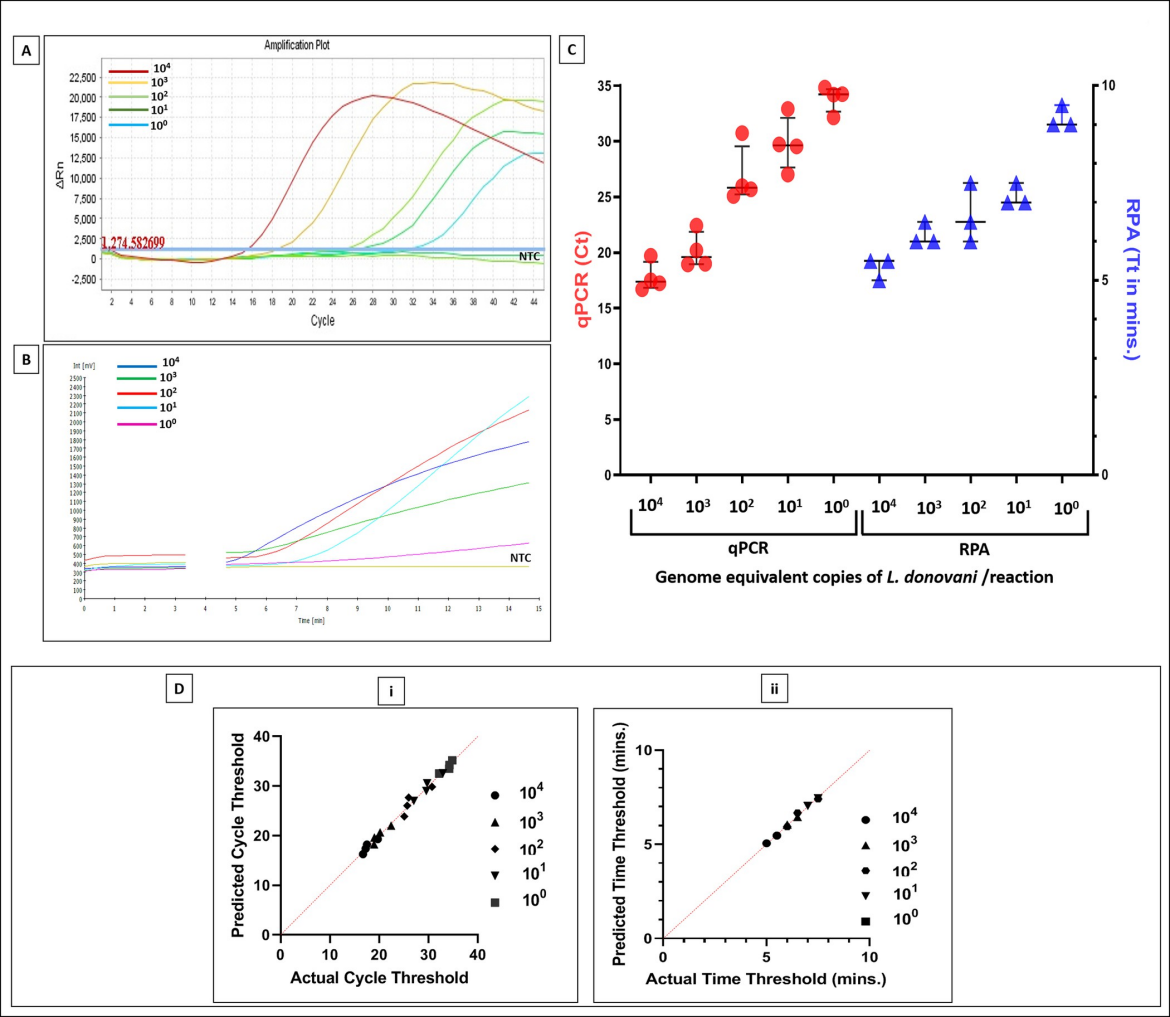
Standard curves for performing qPCR and RPA. **A & B.** Representative amplification curves of qPCR (**A**) and RPA (**B**) runs using serial dilutions (10^4^−10° parasites) of molecular standard derived from a *L*. *donovani* strain together with a non-template control (NTC). Curves were generated for cycle threshold, Ct (cycle number) and time threshold, Tt (minutes) by ABI StepOne software v2.3 and ESEquant tubescanner software, respectively. The drop or gap in RPA (**B**) for the fluorescence signal after three minutes was due to the mixing step, which is necessary to produce a homogenous RPA reaction to increase the assay’s sensitivity as described in materials and methods. **C**. Scatterplots from qPCR (●) and RPA (▲) showing cycle threshold, Ct and time threshold Tt, respectively; the horizontal bar indicates the median (IQR) values. **D**. The quantile-quantile (Q-Q) plot between predicted and actual Ct (**i**) and Tt (**ii**).

The qPCR and RPA assay were initially performed with 10^4^−10^0^ *L*. *donovani* parasites (at least thrice in duplicates) and amplification curves were generated by ABI StepOne v2.3 real time PCR software and ESEquant Tube Scanner software (**Fig 2A and 2B**), respectively. The corresponding threshold cut-offs were set at a Ct >30 for qPCR and Tt >10 minutes for RPA (**Fig 2C**). The addition of blood did not inhibit the reaction. The limit of detection (LOD) was 1.0 genome equivalent copy of parasite(s)/reaction for both qPCR and RPA (**Fig 2C**), and a satisfactory coefficient of variation (CV %) was obtained in both assays (**Table 3).** Both assays showed a significant Pearson rank correlation (r = 0.82, p<0.01) and Spearman rank correlation (r = 0.81, p<0.01). The quantile-quantile (Q-Q) plot for Ct and Tt for 10^4^−10^0^ *Leishmania* standards was performed; it was normally distributed as confirmed by Shapiro-Wilk test with a W value of 0.85, 0.84, 0.76, 0.95, 0.82 for Ct and 0.75, 0.75, 0.96, 0.75, 0.75 respectively for Tt (**Fig 2D, i, ii**). The p values corresponding to Ct W values for 10^4^−10^0^ *Leishmania* standards were 0.22, 0.20, 0.24, 0.72 and 0.16 respectively. Similarly, for Tt the p values were 0.29, 0.21, 0.63, 0.32 and 0.19 respectively. All p values for Ct and Tt were greater than the alpha level (0.05), indicating normal distribution of data sets, accepting the null hypothesis.

**Table 3:** Reproducibility of detection of *Leishmania donovani* by qPCR vs. RPA.

No. of parasites	Ct in cycle number (CV %)	Tt in minutes (CV %)
10^4^	17.79 ± 1.32 (7.44)	5.33 ± 0.28 (5.41)
10^3^	20.14 ± 1.62 (8.07)	6.16 ± 0.28 (4.68)
10^2^	26.87 ± 2.6 (9.69)	6.70 ± 0.76 (11.46)
10^1^	29.79 ± 2.41 (8.11)	7.20 ± 0.28 (4.02)
10°	33.85 ± 1.16 (3.45)	9.20 ± 0.29 (3.14)

A standard curve using *L*. *donovani* parasites ranging from 10^4^−10^0^ was performed by qPCR and RPA as described in materials and methods. All data are expressed in mean ± SEM and CV in percentage; Ct: cycle threshold in qPCR, Tt: time threshold in RPA, CV %: inter assay coefficient of variation, NA: not applicable

### Diagnosis of VL and PKDL by qPCR in comparison with RPA

**Fig 3:**
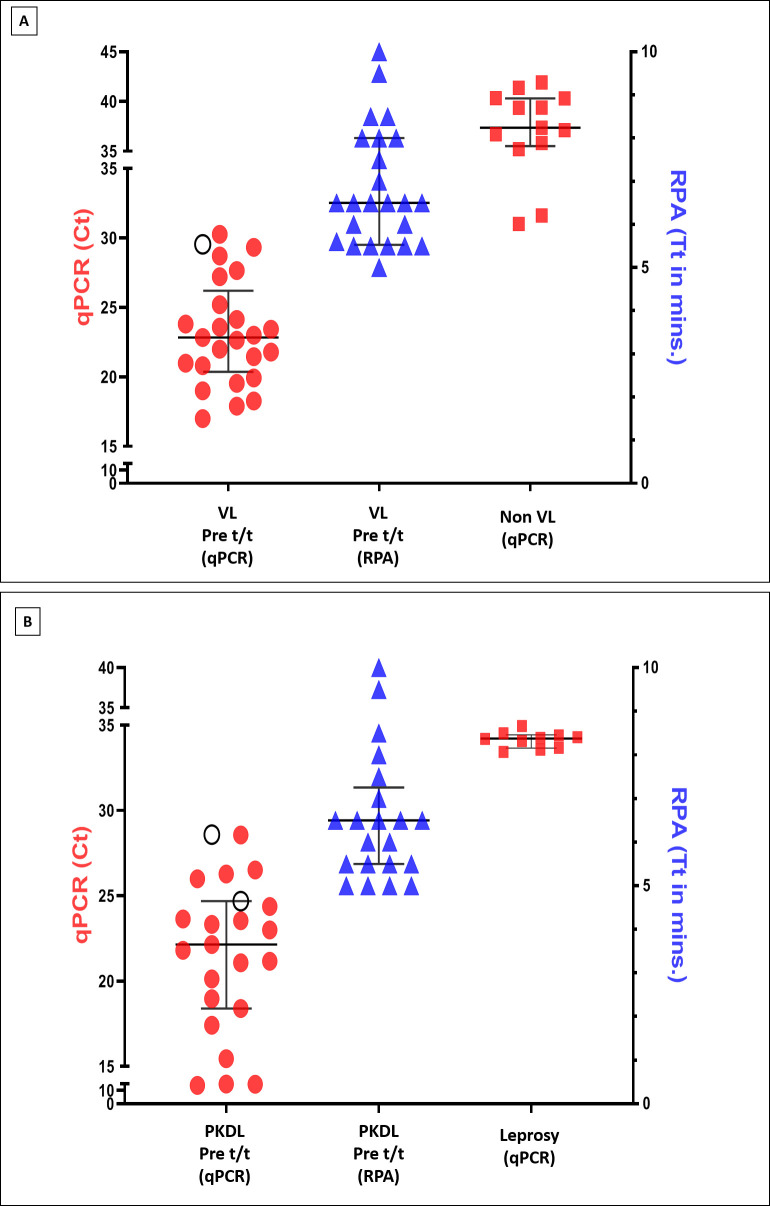
Diagnostic performance of qPCR *vis-à-vis* RPA assays in VL and PKDL cases. **A. Diagnosis of patients with VL in qPCR vs. RPA assays**. Scatterplots from qPCR (●) and RPA (▲) showing cycle threshold (Ct) and time threshold (Tt) performed in peripheral blood sourced from patients with VL (naïve cases, n = 25) and non VL cases (n = 14, ■), as described in materials and methods. Each horizontal bar represents the median (IQR) value; (○) represents a qPCR positive sample that was not detected by RPA. The RPA negative sample had a Tt >10 minutes and therefore it could not be displayed. **B. Diagnosis of patients with PKDL in qPCR *vis-à-vis* RPA assays**. Scatterplots from qPCR (●) and RPA (▲) showing cycle threshold (Ct) and time threshold (Tt) in patients with PKDL (n = 23, naïve cases); leprosy cases (n = 10, ■) served as negative controls to check cross-reactivity. Each horizontal bar represents the median (IQR) value; (○) represents qPCR samples not detected by RPA. The RPA negative samples had a Tt >10 minutes, therefore data could not be displayed.

The parasite load was quantified in naïve patients with VL (n = 25, Group 1, **Fig 1A**) by both assays, and RPA demonstrated concordance in 24/25 qPCR positive cases (**Fig 3A**), the median (IQR) Ct value being 22.83 (20.36–30.26) and median Tt being 6.50 (5.25–8.00) min. (**Fig 3A**). A moderately positive Pearson rank correlation (r = 0.50, p<0.01) and Spearman rank correlation (r = 0.44, p<0.01) was observed between Ct and Tt. The median Ct and Tt for the non VL group was 37.36 (35.52–40.33) and >10 mins respectively.

In case of PKDL, at disease presentation, RPA assay demonstrated concordance with qPCR in 21/23 qPCR positive cases (**Fig 3B**); the median (IQR) Ct was 22.14 (18.39–24.69) and the median Tt was 6.50 (5.50–7.25) minutes (**Fig 3B**). The non VL and leprosy cases tested negative by both assays, confirming no cross-reactivity (**Fig 3A and 3B**). Importantly, there was a strong positive Pearson rank correlation (r = 0.82, p<0.0001) and Spearman rank correlation (r = 0.89, p<0.0001) between Ct and Tt, which was comparable with results obtained using molecular standards.

### Assessment of diagnostic sensitivity and specificity by qPCR with respect to RPA

The sensitivity and specificity of a diagnostic assay refers to the detection of true positive cases and true negative cases respectively. This was calculated for RPA keeping the qPCR as the gold standard. As the RPA assay detected 24/25 VL cases (Group 1, **Table 1**) and demonstrated no false positivity, it translated into 96% sensitivity and 100% specificity. In case of PKDL, 21/23 cases (Group 1, **Table 2**) were detected by RPA, and there was no false positivity, translating into a sensitivity and specificity of 91.3 and 100%, respectively.

### Evaluation of prognostic potential of qPCR in comparison with RPA

Liposomal Amphotericin B (LAmB) and Miltefosine are the ‘drugs of choice’ for patients with VL and PKDL, respectively (http://nvbdcp.gov.in/Doc/Guidelines-Diagnosis-Treatment-KA.pdf, accessed on 10^th^ May, 2022). In VL treated cases (n = 15, Group 2, **Fig 1A and Table 1**), the parasite load was measured by both assays upon completion of treatment, at two time points, either at (i) end of treatment, just prior to hospital discharge, <7 days or (ii) at ≥6 months follow-up. In terms of treatment, the majority of patients received LAmB (n = 13) whereas only 2/15 patients received Miltefosine (**Table 4)**. In both assays, 14 patients demonstrated complete clearance of parasites i.e. qPCR and RPA negative, exhibiting 100% concordance (**Table 4**). There was only one case that received LAmB and returned ≥6 months post treatment. This case tested positive by both assays, and was accordingly considered as ‘relapse’ (**Table 4**). The parasite load, in terms of Ct and Tt for this ‘relapse’ case of VL was 24.76 and 8.00 mins respectively.

**Table 4:** Performance analysis of qPCR and RPA assays for VL following completion of treatment.

Group 2 [n = 15]	Post treatment (Upon hospital discharge, <7days)	Post treatment (Follow-up, ≥6months)
LAmB; n = 13	n = 4 qPCR–ve: 4/4 RPA–ve: 4/4	n = 9 qPCR–ve: 8/9 RPA–ve: 8/9
Miltefosine; n = 2	n = 1 qPCR–ve: 1/1 RPA–ve: 1/1	n = 1 qPCR–ve: 1/1 RPA–ve: 1/1

The number of ‘true negative’ and ‘true positive’ cases detected by RPA was 14 and 1 respectively, in VL cases, Group 2 (post treatment). Similarly, the ‘false negative’ and ‘false positive’ were both 0 (**Table 5**). Therefore, the cure PPV(%) and NPV(%) for RPA was determined as 100%. In another perspective, the RPA was negative in all 14/14 qPCR negative cases and positive in one qPCR positive case, indicating 100% sensitivity or detection accuracy.

**Table 5:** Determination of prognostic positive and negative predictive values of RPA vs. qPCR in cases of VL following completion of treatment (n = 15).

Status	RPA -ve	RPA +ve
**Cured, qPCR -ve**	14	0
**Non cured, qPCR +ve**	0	1

Peripheral blood was collected from patients with VL upon completion of treatment and was evaluated by RPA as described in materials and methods, the qPCR being the gold standard. Patients were considered ‘cured’ when they were qPCR -ve and ‘non-cured’ when they were qPCR positive, VL: visceral leishmaniasis.

Skin biopsies from lesional sites of patients with PKDL (n = 41, Group 2, **Fig 1B and Table 2**) who received Miltefosine (daily for 12 weeks, 50 mg p.o. if <25 kg b.w., and 100 mg p.o. if >25 kg b.w.) were collected at two time points (**Table 6**) i.e., (i) on completion of treatment at 12 weeks (n = 8) or (ii) at any time point, ≥ 6 months following completion of treatment (n = 33). These patients were grouped on the basis of their parasite clearance as (a) cured i.e. qPCR negative or (b) ‘non cured’ i.e. qPCR positive. Accordingly, in the 8 cases examined on completion of treatment at 12 weeks, 4/8 cases were negative by qPCR, hence considered ‘cured’; while 4/8 (50%) cases remained positive, indicating ‘non cured’ (**Table 6**). In these 8 cases, RPA was negative for the 4/4 ‘cured’ cases, while in the 4 ‘non cured’ cases, it was positive in only one (**Table 6**). The median Ct for the 4 ‘non cured’ cases was 27.41 (24.14–28.85).

**Table 6:** Performance analysis of qPCR and RPA assays for PKDL following completion of treatment.

Group 2 [Total:41]	Post treatment (End of 12 weeks)	Post treatment (Follow-up, ≥6months)
Miltefosine; n = 41	n = 8 qPCR–ve: 4/8 RPA–ve: 7/8	n = 33 qPCR–ve: 30/33 RPA–ve: 30/33

In patients of Group 2 (**Table 2**) who presented at any time point, ≥ 6 months following completion of treatment (n = 33, **Table 6**), both assays confirmed complete parasite clearance in 30/33 cases, and only 3/33 remained positive (**Table 6 and 7)**. Accordingly, the 30/33 negative cases were considered as ‘cured’ and the 3/33 positive cases as ‘non-cured’. The median Ct and Tt value of the ‘non-cured’ cases was 22.73 (16.56–23.44), and 7.50 (5.00–1.00) min. respectively. Therefore, irrespective of the time points, the sensitivity or detection concordance of RPA with respect to qPCR was 92.7% (38/41) and discordance was 7.3% (3/41). The number of ‘true negative’ and ‘true positive’ detections by RPA was 34 and 4 respectively, while the ‘false negative’ and ‘false positive’ was 3 and 0 respectively (**Table 7**). Accordingly, the prognostic performance of RPA indicated a cure PPV(%) and NPV(%) of 91.8% and 100% respectively.

**Table 7:** Determination of prognostic positive and negative predictive values of RPA vs. qPCR in patients with PKDL following completion of treatment (n = 41).

Status	RPA -ve	RPA +ve
**Cured** **qPCR -ve**	**34**	**0**
**Non cured** **qPCR +ve**	**3**	**4**

Skin biopsies collected from patients with PKDL were evaluated by qPCR and RPA as described in materials and methods following completion of treatment with Miltefosine, either (i) at the end of 12 weeks (n = 8) or (ii) ≥6 months after completion of treatment (n = 33). The patients were grouped as (a) cured i.e. qPCR -ve or (b) non-cured cases i.e. qPCR +ve. PKDL: post kala-azar dermal leishmaniasis.

## Discussion

The current elimination programme of kala-azar in South Asia is aligned with the global and regional strategies of WHO [visceral leishmaniasis elimination: India gears-up to overcome last-mile challenges, https://www.who.int/news/item/29-07-2021-visceral-leishmaniasis-elimination-india-gears-up-to-overcome-last-mile-challenges, accessed on 10^th^ May, 2022]. The proposed critical gaps that need urgent attention include (a) diagnostics, (b) monitoring and evaluation, (c) access and logistics and (d) advocacy and funding [26]. WHO has reiterated that to achieve the 2030 targets for neglected tropical diseases, accurate field applicable diagnostics are imperative [6]. In PKDL, reaching this end goal of detecting and monitoring parasite load in new cases or relapses respectively is hampered by the patient’s poor healthcare seeking behaviour. With active field surveillance, the scenario has dramatically improved, but there still exists an unacceptable time lag between suspecting a case of PKDL in the field and reaching a confirmatory laboratory diagnosis. In this light, establishment of a rapid and specific molecular field applicable tool is essential, especially in resource-limited VL endemic areas.

In this study, the RPA was evaluated using clinical samples of VL and PKDL (**Fig 1, Tables 1 and 2**) cases at disease presentation (Group 1, **Tables 1 and 2**), and at various time points after completion of treatment (Group 2, **Tables 1, 2, 4, 5, 6, and 7**). Since establishment of the isothermal amplification, the RPA has been applied for monitoring of viral and parasitic infections with considerable success [12–22, 27–29]. However, application of this RPA assay as a monitoring tool after treatment in patients with VL and PKDL was established in this study.

The RPA effectively amplified DNA sourced from a *L*. *donovani* reference strain *vis-à-vis* qPCR and demonstrated a strong correlation between threshold time and parasite load (**Fig 2 and Table 3**). Following the initial testing with a *L*. *donovani* standard, a diverse study population was applied and a strong sensitivity and specificity was achieved. The diagnostic sensitivity of RPA was re-confirmed in naïve cases of VL and PKDL (**Fig 3**) and was in concordance with previous studies [21, 25]. In some cases of VL, the Ct values were close to the cut off value of 30, perhaps attributable to the disease being fatal, and patients more likely to seek early treatment (**Fig 3A**). In such borderline cases, the clinical features along with an additional validation e.g. the rK39 strip test or ITS-1 PCR may be necessary. In contrast, PKDL cases had a longer duration of disease (**Table 2**), and this accounted for fewer cases having Ct values close to the cut off (**Fig 3B**).

In this study, RPA is used as a monitoring tool for parasite load after treatment and to understand the efficacy of its performance with respect to gold standard qPCR. Accordingly, the case definitions of these patients recruited after treatment as cured/non-cured were determined by the results of qPCR being negative/positive respectively. Finally, these were tested by RPA to delineate whether the case definitions are same when compared with qPCR. Additionally, this study was limited in that there was no case that was qPCR negative at recruitment, and later on longitudinal monitoring became qPCR positive. Therefore, the effectiveness of RPA to identify cases that were ‘cured’ or predict relapses remains an unanswered yet pertinent question.

In the field, a diagnostic dilemma may arise between PKDL and leprosy [30] and therefore, assay specificity of RPA was very important (**Fig 3**). Furthermore, the assay specificity has been confirmed with other *Leishmania* species by RPA, detecting *L*. *major*, *L*. *aethiopica and L*. *infantum*, but did not identify *L*. *tropica*, *L*. *amazonensis*, *L*. *braziliensis* and other cross-reactive pathogens (*Toxoplasma gondii*, *Plasmodium falciparum*, *Plasmodium vivax*, *Salmonella typhi*, *Mycobacterium tuberculosis*) [25], endorsing the assays robustness.

The field applicability along with high sensitivity and specificity of the isothermal amplification-based assays has attracted tremendous interest in diagnostics [31]. Several assays have been developed using the isothermal platform e.g., RPA, LAMP, nucleic acid sequence-based assay (NASBA) in sand fly specimens and human leishmaniasis [32–38]. The LAMP assay effectively predicted relapses in PKDL [36]. Although, both LAMP and RPA assays are field applicable, the latter offers several advantages, in terms of faster time to results, simpler primer design, longer target sequence, more tolerance to inhibitors, and dispensation of the heating source. The amplification of DNA in the RPA relies on enzymes and proteins to replace the repetitive cycles of three temperatures (94°C, DNA denaturation; 50–60°C, primer annealing; 72°C extension) necessary in the PCR and the reaction occurs at a constant temperature (40°C). However, till date, the RPA has not been compared with LAMP or any other isothermal amplification techniques.

The introduction of a single infusion of liposomal Amphotericin B (LAmB) for VL proved to be a game changer, and there is currently a need for tool(s) that will support monitoring of chemotherapy and early detection of relapses. In VL, the RPA assay effectively identified complete ‘cure’ and importantly, detected a case of ‘relapse’ (**Tables 4 and 5**). Moreover, this ‘relapse’ case detected by molecular methods corroborated with clinical symptoms as the patient presented with fever and hepatosplenomegaly. The strong cure PPV(%) and NPV(%) confirmed the prognostic potential of RPA for VL. To the best of our knowledge, this is the first study in VL to evaluate prognosis employing the RPA assay and therefore this assay contribute towards the KAEP targeted for 2023. Although the prognostic value of a test is best assessed in a longitudinal study, where a prerequisite is to have paired samples before and after completion of treatment, this assay was performed in a relatively limited number of post-treatment cases as the study period was during the COVID-19 pandemic. Furthermore, in view of a skin biopsy being needed for each evaluation, it was not possible to serially collect biopsies. Therefore, the possible impact of time after treatment on the test could not be evaluated. The currently available prognostic markers in VL and PKDL are serological (IgG3, IgG1), and have shown promise in VL cases for detection of relapse [39], and for assessing therapeutic effectiveness in PKDL [40]. Additionally, serum anti-rK39 antibody levels exhibited promising results with predictive values in VL relapse and development of PKDL [41].

The diagnosis of PKDL has till date been empirically decided, based on clinical features, as the parasite load was not quantified due to non-availability of molecular tools in clinical practice. It is anticipated that the RPA would resolve this limitation, and by supporting clinical trials would allow for availability of better chemotherapy regimens. On a clinical basis, the currently available lesion score was developed for PKDL in Sudan (http://apps.who.int/iris/bitstream/handle/10665/101164/9789241504102_eng.pdf;jsessionid=877F5C1DD6A9187E78DD407E6D9A84EB?sequence=1, accessed on 17^th^ November, 2022). However, as clinical presentations differ, it is difficult to extrapolate this score to the South Asian form, emphasizing the need for development of a disease activity score for the different forms of PKDL.

Presently, Miltefosine, an orally effective antileishmanial drug, has been found to be well tolerated and effective for PKDL [3,42], but requires a prolonged therapy of 84 days which increases the possibility of poor adherence to treatment. Ideally, the cure assessment should be done at least 12 months after completion of treatment. However, as the dropout rate beyond 12 months is very high, the cases were asked to report at any time point ≥ 6 months, and this ranged from 1.50 (1.00–1.85) years. In PKDL, the strong assay concordance and cure PPV vs. NPV% established the RPA’s ability to predict response to treatment (**Tables 6 and 7**). However, the RPA failed to detect 3/4 ‘non-cured’ cases that were qPCR positive (**Tables 6 and 7**). In these 3 cases, the Ct ranged from 27–28, and was near the cut off threshold Ct value of 30, suggestive of a low parasite load, within the range of 10–100 genome equivalent copies of parasite kDNA. Therefore, the RPA would benefit from further fine tuning for detection of cases having a low parasite load. Importantly, these three patients of PKDL were enrolled just at the end of their 12 weeks treatment with Miltefosine, and therefore it is also possible that their low parasite load would be effectively cleared by the host immune system in the succeeding months. Generally, the clinical outcome of cure for PKDL is evaluated by a dermatologist based on near total regression of papules/nodules/macules, no new lesion(s), and considerable regression of macular lesions [4]. However, repigmentation in macular cases can be delayed and accordingly, we propose that on completion of treatment, a case definition of ‘cure’ be established, by including an objective parameter e.g. parasitological clearance along with clinical features. As the elimination of leishmaniasis in South Asia hinges on the longitudinal monitoring of patients with VL and PKDL, a dire requirement is availability of rapid DNA-based assays. In this essence, the portability of RPA is indeed an advantage in clinical settings. The RPA assay exhibited comparable performance with respect to qPCR, and due to the ease of field applicability, is well-positioned to become a relevant molecular tool as a potential point of care management in PKDL. However, the experimental setup in this study provides incremental evidence, but does not permit a conclusion that the RPA assay is a ‘test of cure’. This necessitates assessment of RPA with longitudinal patient follow up for exploring its potential as ‘point-of-cure’ tool and predict relapses. In view of the RPA assay offering the opportunity to be an affordable molecular platform for leishmaniasis, it can accelerate overcoming the last mile hurdles pertaining to the ongoing leishmaniasis elimination program.

## Supporting information

S1 Fig Representative images of patients with polymorphic and macular PKDL at disease presentation (A) and following completion of treatment (B).
(TIF)

S2 Fig Semi-quantitative PCR analysis of the human specific *18s* amplified region of DNA extracted from peripheral blood and skin biopsy randomly sourced from patients with VL and PKDL respectively. Lanes: M: molecular ladder, 100-bp ladder; 1: PCR control (nuclease-free water); 2–6: VL (n = 5, qPCR and RPA negative samples), 7–11: PKDL (n = 5, qPCR and RPA negative samples). The amplified PCR products were run with 2% agarose gel electrophoresis.
(TIF)
