# Supplementary figures and images for: Evaluation of Recombinase Polymerase Amplification assay for monitoring parasite load in patients with kala-azar and post kala-azar dermal leishmaniasis
Source: PLoS Negl Trop Dis. 2023 Apr 19;17(4):e0011231. doi: 10.1371/journal.pntd.0011231 (PMC10115299; doi:10.1371/journal.pntd.0011231)

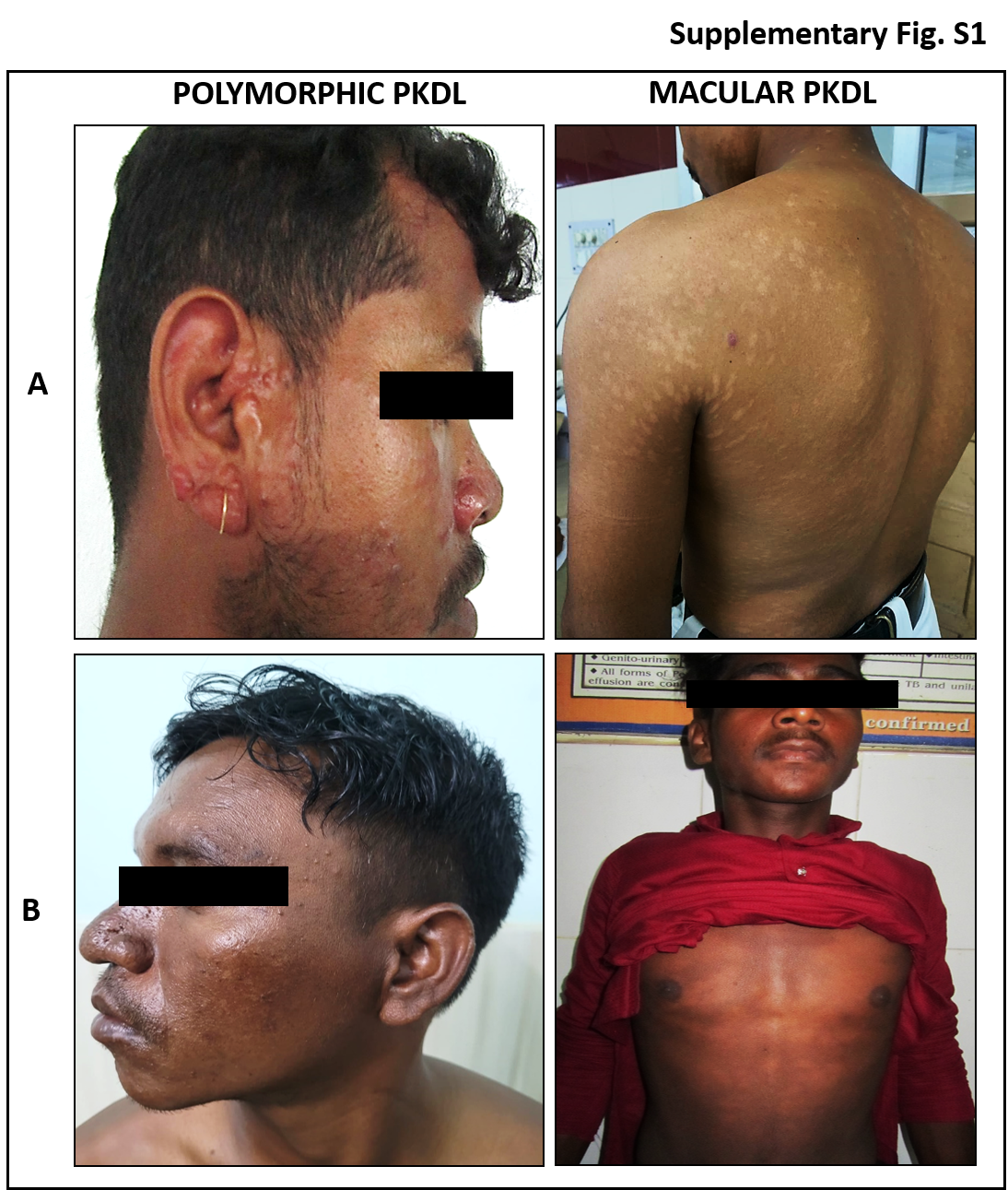

Supplement: S1 Fig — Representative images of patients with polymorphic and macular PKDL at disease presentation (A) and following completion of treatment (B). (TIF) [file pntd.0011231.s001.tif]

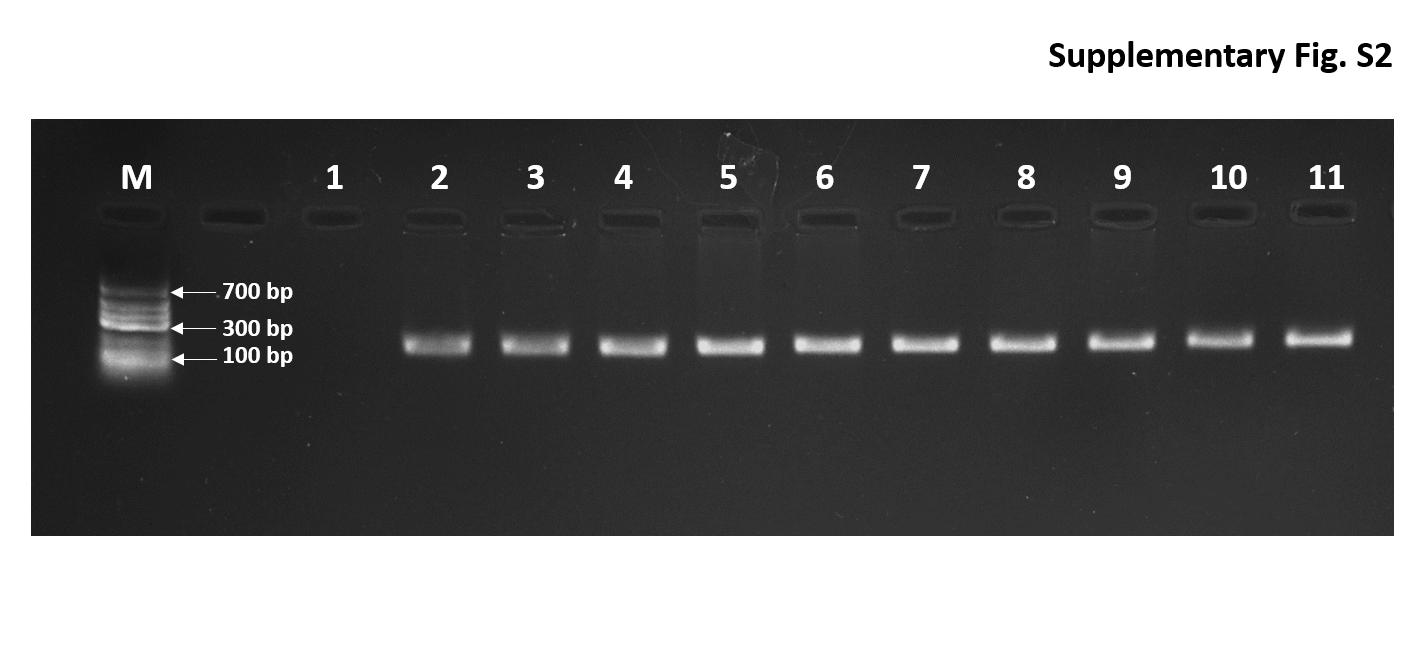

Supplement: S2 Fig — Lanes: M: molecular ladder, 100-bp ladder; 1: PCR control (nuclease-free water); 2–6: VL (n = 5, qPCR and RPA negative samples), 7–11: PKDL (n = 5, qPCR and RPA negative samples). The amplified PCR products were run with 2% agarose gel electrophoresis. (TIF) [file pntd.0011231.s002.tif]
